# Clinical characteristics and perinatal outcome of fetuses with ventriculomegaly

**DOI:** 10.1007/s00404-024-07599-8

**Published:** 2024-06-26

**Authors:** Ebru Alici Davutoglu, Gorkem Arica, Nazli Ece Sahin, Ayse Kalyoncu Ucar, Ibrahim Adaletli, Zekeriyya Mehmet Vural, Riza Madazli

**Affiliations:** 1 Department of Obstetrics and Gynecology, Division of Perinatology, Cerrahpasa Medical Faculty, Istanbul University-Cerrahpasa, Kocamustafapasa, Istanbul, Turkey; 2 Department of Obstetrics and Gynecology, Cerrahpasa Medical Faculty, Istanbul University-Cerrahpasa, Istanbul, Turkey; 3 Department of Pediatric Radiology, Cerrahpasa Medical Faculty, Istanbul University-Cerrahpasa, Istanbul, Turkey; 4 Department of Neonatology, Cerrahpasa Medical Faculty, Istanbul University-Cerrahpasa, Istanbul, Turkey

**Keywords:** Fetal magnetic resonance imaging, Fetal cerebral ventriculomegaly, Perinatal outcome, Prenatal diagnosis

## Abstract

**Purpose:**

To assess the incidence of associated structural anomalies, chromosomal/genetic abnormalities, infections, and perinatal outcomes of fetuses with ventriculomegaly (VM), also to evaluate the role of fetal magnetic resonance imaging (MRI) in detecting associated intracranial anomalies.

**Methods:**

Retrospective cohort study of 149 prenatally diagnosed pregnancies with fetal VM. VM was classified as mild (Vp = 10–12 mm), moderate (Vp = 12.1–15 mm), and severe (Vp > 15 mm). Fetal MRI was performed to 97 pregnancies.

**Results:**

The incidences of an associated CNS, non-CNS, chromosomal anomaly, genetic abnormality and fetal infection were 42.3%, 11.4%, 6.1%, 2.1% and 1.3%, respectively. Fetal MRI identified additional CNS anomalies in 6.7% of cases, particularly in severe VM. The incidences of perinatal outcomes were 18.8% termination of pregnancy, 4% intrauterine and 8.1% neonatal or infant death. The rates of fetuses alive at > 12 months of age with neurological morbidity were 2.6%, 11.1% and 76.9% for mild, moderate and severe isolated VM, respectively.

**Conclusion:**

The prognosis of fetuses with VM mostly depends on the severity and the associated anomalies. Mild to moderate isolated VM generally have favorable outcomes. Fetal MRI is particularly valuable in fetuses with isolated severe VM.

## What does this study add to the clinical work

**Table:** 

This study emphasizes that isolated mild-moderate VM may be associated with neurodevelopmental delay and a long-term poor neurological outcome. Fetal MRI is particularly valuable in cases of severe ventriculomegaly.	

## Introduction

Fetal ventriculomegaly (VM) is defined as enlargement of the atrium of the lateral cerebral ventricles and it is the most common central nervous system (CNS) abnormality detected by prenatal US [1]. The incidence is approximately 1% and it occurs in up to 2 per 1000 births [1, 2]. The measurement of the atrial diameter (Vp) remains stable between 15 and 40 weeks’ gestation [3] The classification of VM varies but is generally classified into either mild (10–12 mm), moderate (12.1–15 mm) and severe VM (> 15 mm) or mild (10–15 mm) and severe VM (> 15 mm) [4, 5].

The prognosis of fetal VM is widely variable, which makes prenatal counselling quite challenging in clinical practice. Fetal VM is not a diagnosis but rather a sonographic sign. The prognosis depends on whether it is combined with structural abnormalities, especially CNS anomalies, chromosomal aberrations, congenital infections and the progression of the ventricular dilation [4, 6]. Following confirmation of the diagnosis, a thorough evaluation including complete examination of the fetal anatomy, detailed neurosonographic assessment, preferably using the transvaginal approach if possible; consideration of fetal magnetic resonance imaging (MRI), amniocentesis for karyotype and chromosomal microarray (CMA), and investigations for fetal infections should be performed [6, 7].

The reported incidence of additional CNS and non-CNS anomalies ranges between 10 and 85% and are more common in severe VM [6, 8, 9]. Fetal MRI has an increasing role in the investigation of underlying problems and especially improves the diagnosis and characterization of accompanying CNS anomalies. A recent meta-analysis reported that about 10% of fetuses presenting with isolated mild or moderate VM on ultrasound have associated CNS anomalies detected by fetal MRI [10]. Chromosomal abnormalities reported in the literature varies between 2 and 15%, depending on the study population, atrial width, and the presence of additional structural malformations [5, 11]. Moreover, copy number variants (CNVs) are increasingly being recognized as a significant contributor to the poor neurodevelopmental outcome in fetuses with isolated VM [12]. The prevalence of infections in fetuses with VM is about 1.4% with the most common infective agents being cytomegalovirus (CMV) and toxoplasmosis [6].

The objective of this study was to present the experience of fetal VM in a tertiary center. We aimed to determine the incidence of associated CNS and non-CNS anomalies, chromosomal/genetic abnormalities, infectious causes, and perinatal outcomes according to the type of VM. And, to identify the rate of additional CNS anomalies detected exclusively on prenatal MRI in fetuses diagnosed with isolated VM on ultrasound.

## Methods

This was a retrospective cohort study of 149 prenatally diagnosed pregnancies with fetal VM at Cerrahpasa Medical Faculty, Department of Obstetrics and Gynecology, Division of Perinatology in a period between January 2014 and February 2023. The study was approved by the local Ethical Committee (E-83045809-604.01.01-728474). Neural tube defects, twin pregnancies and cases with incomplete follow-up were excluded. The ultrasound equipment used during the study period was Voluson E10 (GE Healthcare, Zipf, Austria) machines with 5 or 3.5 MHz transducers. All women had undergone an expert ultrasound assessment, including multiplanar neurosonography using transabdominal and/or, when feasible, transvaginal approach, as suggested by the ISUOG guidelines [13]. The atrium of the LV measured in the axial transventricular plane at the level demonstrating the frontal horns and cavum septi pellucidi (CSP), in which the cerebral hemispheres are symmetrical in appearance. The calipers were positioned on the internal margin of the medial and lateral walls of the atria, at the level of the parietal-occipital groove and glomus of the choroid plexus [4].VM was classified into mild, moderate, and severe groups as follows; mild Vp = 10–12 mm, moderate Vp = 12.1–15 mm and severe Vp > 15 mm. The fetuses were also classified as "isolated ventriculomegaly" (IVM) if no associated anomaly was detected at the initial ultrasonography with negative findings in TORCH screening and karyotype examinations. In each case, a thorough sonographic evaluation of fetal anatomy was performed, and additional structural anomalies were recorded. Fetal MRI was performed to 97 pregnancies with fetal VM with 1.5 T Magnetom Avanto System (Siemens, Germany). These women did not have any known or suspected contraindications to MRI and agreed to provide written consent after full explanation. T2-weighted sections were taken in 3 orthogonal planes as axial, coronal, and sagittal with a section thickness of 3–4 mm. T1-W images were routinely taken to detect bleeding, fat or calcification in axial plane. Fetal MRI images were evaluated and reported by two of the authors (IA, AKU).

Maternal age, gestational age at the time of diagnosis, karyotype results, CNS and non-CNS anomalies, gestational week at delivery, birth weight, postnatal definitive diagnosis, infections, chromosomal and genetic abnormalities were analyzed. All cases were classified into five groups according to pregnancy outcome: termination of pregnancy (TOP), intrauterine death, neonatal and infant death, and survivors. The decision of TOP was made by the official ‘Termination of Pregnancy Council’ of our faculty according to national laws. Neonatal death was defined as death within the first 28 days of life, and infant death was defined as death within the first year. The median age of the surviving infants was 4.5 ± 2.1 year (range, 1–9 years) at the time of the study. Data on surviving infants were obtained by telephone interviews with the parents. Major neuromotor abnormalities and ventriculoperitoneal shunt operations due to VM was defined as morbidity.

The data were analyzed using Statistical Package for Social Sciences software version 20.0 for Windows (SPSS Inc., Chicago, IL). Categorical variables were reported as frequencies and percentages, with mean and standard deviation reported for continuous variables. Parametric data were appraised with an independent two-sample *t*-test and one-way ANOVA. Nonparametric data were compared using the Kruskal–Wallis and Mann–Whitney *U*-test.

## Results

The general characteristics and the perinatal outcomes of the study population are demonstrated in Table 1. The mean maternal age was 28.8 ± 4.8 years. The mean gestational age at the first examination in our center was 24.5 ± 5.3 weeks and 53% were ≤ 24 weeks’ gestation. Of the included cases, 63.8% were affected by bilateral VM. VM was mild (10.0–12 mm) in 48.3%, moderate (12.1–15 mm) in 19.5% and severe (> 15 mm) in 32.2% of the cases. The incidence of an associated structural anomaly was 53.7%, of which 42.3% was CNS and 11.4% was non-CNS anomalies. The incidences of chromosomal anomaly, genetic abnormality and fetal infection of the study group were 6.1%, 2.1% and 1.3%, respectively. Concerning the outcomes of the total population; 28 (18.8%) cases had TOP, 6 (4%) cases had intrauterine, and 12 (8.1%) cases had neonatal or infant death. Of the 115 live-born babies, mean gestational age at delivery and mean birthweight was 37.9 ± 2.1 weeks and 3134 ± 540 g, respectively. Of the 103 babies alive at > 12 months of age, 32 (31.1%) were with and 71 (68.9%) were without neurological morbidity.

**Table 1:** The clinical characteristics and the perinatal outcomes of the study population

*N*	149
Maternal age (years)	28.8 ± 4.8
Gestation age at examination (weeks)	24.5 ± 5.3
< 20 weeks	23, 15.4
20–24 weeks	56, 37.6
25–32 weeks	59, 39.6
> 32 weeks	11, 7.4
Ventricular width (mm)	
10–12	72, 48.3
12.1–15	29, 19.5
> 15	48, 32.2
Laterality	
Bilateral VM	95, 63.8
Unilateral VM	54, 36.2
Associated structural anomalies	80, 53.7
Central nervous system	63, 42.3
Non-central nervous system	17, 11.4
Chromosomal anomaly	9, 6.1
Genetic abnormality	3, 2.1
Fetal infection	2, 1.3
Gestational age at birth (weeks) (*N* = 115)	37.9 ± 2.1
Birthweight (grams) (*N* = 115)	3134 ± 540
Termination of pregnancy	28, 18.8
Intrauterine death	6, 4
Neonatal and infant death	12, 8.1
Alive at > 12 months of age	103, 69.1
With morbidity	71/103, 68.9
Without morbidity	71/103, 68.9

Data are expressed as mean ± *sd* and *n*,%

Distribution of associated structural, chromosomal and genetic anomalies according to the type of VM is illustrated in Table 2. There was no significant difference between the incidences of CNS and non-CNS anomalies, chromosomal and genetic abnormalities between the VM groups (*p* > 0.05). There were four chromosomal anomalies (trisomy 21, trisomy 22, triploidy and Koolen de Vries syndrome (17 q 21.31,664 KB del of KANSL 1)) in the mild, one chromosomal anomaly (partial trisomy 8) in the moderate and four chromosomal anomalies (trisomy 21, 4 *p* microdeletion, increase in the *p* arm of chromosome 15, 46 –*t*(6;17)(*p*25:*q*23)) in the severe VM group. There were three genetic abnormalities identified in our series: two in the mild (Zellweger syndrome and nonketotic hyperglycinemia) and one in the severe (Walker Warburg syndrome) VM group. There were two fetal infections confirmed by amniotic fluid PCR analysis: one case with toxoplasma had moderate VM, periventricular nodule and hyperechogenic bowel and the other case with CMV had severe VM, periventricular calcification and microcephaly. Both fetuses with congenital infections had TOP.

**Table 2:** Distribution of associated structural, chromosomal and genetic anomalies according to the type of ventriculomegaly

	Mild VM	Moderate VM	Severe VM
*N*	72	29	48
Central nervous system anomalies	23, 31.9	13, 44.8	27, 56.3
Corpus callosum agenesis	13	6	11
Dandy Walker	5	1	3
Intracranial hemorrhage	3	2	6
Intracranial cyst	1	2	–
Cerrebelum hypoplasia	1	–	1
Semilober holoprosencephaly	–	1	1
Hemimegalencephaly	–	–	1
Lissencephaly	–	–	1
Schizencephaly	–	–	1
Hydranencephaly	–	–	1
CSP agenesis	–	1	–
Walker Warburg syndrome	–	–	1
Non-central nervous system anomalies	8, 11.1	6, 20.7	3, 6.3
Infection	–	1, 3.4	1, 2.1
Chromosomal anomalies	4, 5.6	1, 3.4	4, 8.3
Genetic abnormalities	2, 2.8		1,2.1

Data are expressed as *n* or *n*,%

*CSP* cavum septum pellucidum, *VM* ventriculomegaly

Associated CNS anomalies were detected in 23 (31.9%), 13 (44.8%) and 27 (56.3%) of the fetuses with mild, moderate and severe VM, respectively (Table 2). Detected CNS malformations were corpus callosum agenesis, intracranial hemorrhage, Dandy-Walker malformation, intracranial cyst, cerebellar hypoplasia, semilober holoprosencephaly, hemimegalencephaly, lissencephaly, schizencephaly, hydranencephaly, Walker Warburg syndrome and CSP agenesis in 30 (48.4%), 11 (17.7%), 9 (14.5%), 3 (4.8%), 2 (3.2%), 2 (3.2%), 1 (1.6%), 1 (1.6%), 1 (1.6%), 1 (1.6%), 1 (1.6%) and 1 (1.6%) of the VM cases, respectively. Fetal MRI was performed in 97 (65.1%) of the fetuses with VM in the present study. The mean gestational age at fetal MRI examination was 27.1 ± 4.6 weeks (range, 20–36 weeks). Rates of additional anomalies detected on prenatal MRI are shown in Table 3. There were 10 (6.7%) additional CNS anomalies diagnosed by fetal MRI in our series. Incidences of additional anomalies detected on prenatal MRI were 4.2%, 3.4% and 12.5% for mild, moderate and severe VM, respectively. Fetal MRI was more effective in diagnosing additional CNS anomalies in fetuses with severe VM. Of these additional anomalies, 3 (10%) of the 30 CCA, 6 (54.5%) of the 11 intracranial hemorrhage and 1 Walker Warburg syndrome were diagnosed by fetal MRI.

**Table 3:** Additional anomalies diagnosed on prenatal magnetic resonance imaging

	Mild VM	Moderete VM	Severe VM
*N*	72	29	48
Gestational age at examination (weeks)	26.2 ± 4.6	29.1 ± 4.7	27.1 ± 4.6
Fetal MRI	47, 65.2	19, 65.5	31, 64.5
Additional anomaly	3, 4.2	1, 3.4	6, 12.5
Complete corpus callosum agenesis	–	–	2
Partial corpus callosum agenesis	1	–	–
Hemorrhage	2	1	3
Walker Warburg syndrome	–	–	1

Data are expressed as mean ± *sd* and *n* or *n*,%

*VM* ventriculomegaly

The clinical characteristics and the perinatal outcomes of mild, moderate and severe VM groups according to the additional structural anomalies are shown in Table 4. Incidence of chromosomal anomalies was significantly higher in fetuses with additional structural anomalies than without associated anomalies (8/80, 10% vs 1/69, 1.4%) (*p* = 0.029). Incidence of TOP was significantly higher in fetuses with VM and additional structural anomalies (21/80, 26.3% vs 7/69, 10.1%) (*p* = 0.012). Incidence of surviving babies alive at > 12 months of age without morbidity was significantly lower in fetuses with VM and additional structural anomalies (23/80, 28.8% vs 48/69, 69.6%) (*p* < 0.001). Of the 41 fetuses with mild VM and without additional structural anomalies, 1 (2.4%) had chromosomal and 2 (4.8%) had genetic abnormalities in our study population.

**Table 4:** The clinical characteristics and the perinatal outcomes of mild, moderate and severe ventriculomegaly groups according to the additional structural anomalies

	Mild VM		Moderate VM		Severe VM	
	Without associated anomaly	With associated anomaly	Without associated anomaly	With associated anomaly	Without associated anomaly	With associated anomaly
*N*	41	31	10	19	18	30
Gestational age at birth (weeks)	38.1 ± 1.7	38.1 ± 1.8	38.7 ± 0.9	38.2 ± 1.6	37.7 ± 1.3	37.1 ± 2.8
Birthweight (grams)	3128 ± 448	3031 ± 550	3393 ± 391	3254 ± 531	3171 ± 589	3075 ± 681
Chromosomal anomaly	1, 2.4	3, 9.7	–	1, 5.3	–	4, 13.3
Genetic abnormality Fetal	–	–	1, 10	–	1, 5.6	–
Termination of pregnancy	1, 2.4	13, 41.9	1, 10	4, 21.1	5, 27.8	4, 13.3
Intrauterine death	–	–	–	3, 15.8	–	3, 10
Neonatal and infant death	2, 4.8	2, 6.5	–	2, 10.5	–	6, 20
Alive at > 12 months of age						
With morbidity	1/38, 2.6	3/16, 18.6	1/9, 11.1	4/10, 40	10/13, 76.9	13/17, 76.5
Without morbidity	37/38, 97.4	13/16, 81.4	8/9, 89.9	6/10, 60	3/13, 23.14	4/17, 23.5

Data are expressed as mean ± *sd* and *n*,%

*VM* ventriculomegaly

Perinatal outcomes of isolated mild, moderate and severe VM groups are demonstrated in Table 5. The incidence of isolated VM in our study group was 42.9% (64/149). Of the 64 fetuses with isolated VM, 4 with severe VM opted for TOP and there were no intrauterine and neonatal death. The rates of fetuses alive at > 12 months of age with neurological morbidity were 2.6%, 11.1% and 76.9% for mild, moderate and severe isolated VM, respectively. The incidence of surviving babies with neurological morbidity was significantly higher in fetuses with severe isolated VM than mild and moderate VM (*p* < 0.001). The comparison of perinatal outcomes between the groups related to laterality was also analyzed. Postnatal morbidity and neonatal death were significantly higher in the bilateral ventriculomegaly group compared to the unilateral ventriculomegaly group (*p* = 0.017, *p* = 0.001).

**Table 5:** Perinatal outcomes of isolated mild, moderate and severe ventriculomegaly groups

	Mild VM	Moderete VM	Severe VM
*N*	38	9	17
Termination of pregnancy	–	–	4
Intrauterine death	–	–	–
Neonatal and infant death	–	–	–
Alive at > 12 months of age			
With morbidity	1/38, 2.6	1/9, 11.1	10/13, 76.9
Without morbidity	37/38, 97.4	8/9, 89.9	3/13, 24.1

Data are expressed as mean ±  *sd* and *n*,%

*VM* ventriculomegaly

## Discussion

The mean maternal age and the average gestational age at the first diagnoses of fetal VM cases in our study population are similar to those reported in the literature [5, 8, 14]. In our series, 53% of the fetal VM cases were diagnosed ≤ 24 weeks’ and 7.4% after 32 weeks’ gestation. Joo´ et al. reported that 60% of the prenatal diagnosis in their series was made before the 24th gestational weeks’ [14]. Incidences of mild, moderate and severe VM cases in our study group are also in accordance with other studies [5, 15]. Nearly half of the cases (48.3%) were mild VM in our study population, in a series by Chang et al. the incidence of mild VM was 50.4% [15].

VM has a variety of causes and all fetuses with VM must be very carefully evaluated for its cause and associated structural abnormalities. It is well established that VM is associated with additional structural abnormalities, with a reported incidence up to 50% [5, 9, 15, 16], which was consistent with 53.7% in our series. A significant aggravation of the outcome is recognized in the presence of associated structural malformations of the CNS and/or extra CNS [15, 16]. Incidence of surviving babies alive at > 12 months of age without morbidity was significantly higher in fetuses without associated structural malformations in our study group in accordance with the literature.

In clinical practice, fetal MRI is commonly used as an adjunct to improve diagnostic accuracy for brain abnormalities and can be used to identify subtle CNS anomalies overlooked at the ultrasound [5–7, 14]. Fetal brain MRI has been accepted as a complementary method, however, the indications and further value of this method, have not been fully established [17]. In a recent systematic review, Di Mascio et al. found that, in fetuses with isolated mild and moderate VM, the incidence of CNS anomalies detected exclusively on MRI was 5% when dedicated neurosonography was performed and 16.8% with standard assessment of the fetal brain [10]. In a multicentre, retrospective, cohort study designed by ENSO Working group demonstrated that in fetuses with isolated mild or moderate VM, the incidence of an associated fetal anomaly detected by fetal MRI was 5.4% [18]. In a recent multicentre study, 18.1% of fetuses with severe VM had additional structural anomalies detected by fetal MRI [19]. The most frequent CNS anomalies detected by fetal MRI were cortical development malformations, intracranial hemorrhage and midline anomalies in reported series [10, 18, 19]. In our series, MRI was performed in 65.1% of cases and provided additional information in 4.2%, 3.4% and 12.5% for mild, moderate and severe VM, respectively. The fetal MRI protocol in our department involves performing an MRI scan around the 26th week of gestational age. However, the number of viable fetuses in our cohort was decreased due to termination or in utero death before 28 weeks. Additionally, some patients expressed concerns about fetal MRI and declined to undergo the scan during pregnancy. The most common anomaly detected by fetal MRI was intracranial hemorrhage in our study. We assume that fetal MRI should be considered as a part of the prenatal assessment of fetuses presenting with isolated VM, especially in cases with severe VM.

The incidence of abnormal karyotype associated with VM varies between 2 and 15%, depending on the study population, atrial width, and additional structural malformations [20, 21]. Chromosomal microarray analysis [CMA] is advised to be offered if the chromosome is found to be normal [4]. The incidence of chromosomal abnormality was 6.1% in our study, in accordance with the literature [4]. The incidence of chromosomal abnormality in mild VM without associated anomaly was 2.4% and 13.3% in fetuses with severe VM associated with structural anomaly in our group. The exact relationship between the degree of VM and the incidence of chromosomal abnormalities is not well known. However, severe VM with associated structural anomaly seems to be more likely affected by chromosomal abnormalities [22]. Fetal ventricular enlargement can rarely be the only, non-specific sign of a genetic condition or a metabolic disorder in the absence of additional CNS and non-CNS malformations [16]. We have diagnosed three genetic syndromes with fetal VM and whole exome sequencing [WES] could be offered to diagnose genetic abnormalities in selected cases [23].

The survival rate of isolated mild and moderate VM is reported to be 97–98%, and normal neurodevelopment is expected in more than 90% of these children [24]. The survival rates were 100%, and the incidences of neurological morbidities were 2.6% and 11.1% for mild and moderate VM in our study population. Isolated mild–moderate VM can be associated with neurodevelopmental delay and long-term neurological outcome studies have conflicting results in the literature [20]. Isolated severe VM is associated with poor neurodevelopmental outcome and high incidences of TOP. In a recent meta-analysis, Carta et al. demonstrated that the survival rate was 88% in isolated severe VM and of the surviving babies 18.6% had mild-moderate and 39.6% had severe neurologic, motor, and cognitive impairment [25]. In accordance with the literature, the survival rate was 76.5% and the incidence of neurological morbidity was 76.9% in our isolated severe VM cases.

Despite the comprehensive nature of this study, there are some limitations that should be acknowledged. Firstly, the sample size was relatively small, which may limit the generalizability of the findings to broader populations. Additionally, the rate of diagnostic tests for chromosomal and genetic abnormalities was low. Furthermore, the information collected about postnatal follow-up through phone interviews with parents, particularly regarding the lack of information from pediatric neurology follow-up, may diminish the reliability of our data.

In conclusion, our results suggest that the prognosis of fetuses with VM mainly depends on the aetiology and on the presence of associated abnormalities. Detailed anatomical examination, expert neurosonography, fetal MRI, genetic analysis and investigations for fetal infection should be performed. Fetuses with mild–moderate isolated VM have a favourable outcome. However, the possibility of neurological impairment could not be completely excluded in fetuses with mild–moderate isolated VM.

## Data Availability

The data used to support the findings of this study are available from the corresponding author upon reasonable request.
